# Molecular networks of FOXP family: dual biologic functions, interplay with other molecules and clinical implications in cancer progression

**DOI:** 10.1186/s12943-019-1110-3

**Published:** 2019-12-09

**Authors:** Ju-Ha Kim, Jisung Hwang, Ji Hoon Jung, Hyo-Jung Lee, Dae Young Lee, Sung-Hoon Kim

**Affiliations:** 1 Cancer Molecular Target Herbal Research Lab, College of Korean Medicine, Kyung Hee university, 1 Hoegi-dong, Dongdaemun-gu, Seoul 02447 Republic of Korea; 2 Department of Herbal Crop Research, Rural Development Administration, National Institute of Horticultural and Herbal Science, Eumseong, 27709 Republic of Korea

**Keywords:** FOXP proteins, Molecular networks, Noncoding RNAs, Cellular immunotherapy, Clinical implications and cancer progression

## Abstract

Though Forkhead box P (FOXP) transcription factors comprising of FOXP1, FOXP2, FOXP3 and FOXP4 are involved in the embryonic development, immune disorders and cancer progression, the underlying function of FOXP3 targeting CD4 + CD25+ regulatory T (Treg) cells and the dual roles of FOXP proteins as an oncogene or a tumor suppressor are unclear and controversial in cancers to date. Thus, the present review highlighted research history, dual roles of FOXP proteins as a tumor suppressor or an oncogene, their molecular networks with other proteins and noncoding RNAs, cellular immunotherapy targeting FOXP3, and clinical implications in cancer progression.

## Background

Cancer still remains a major factor of human deaths worldwide to date [1]. It is well documented that epigenetic and genetic alterations including transcription factors, growth factors, cytokines, chemokines and proteases are critically involved in cancer progression under specific microenvironment [2]. As one of transcription factors Forkhead box P (FOXP) family consist of FOXP1 (3p14.1), FOXP2 (7q31), FOXP3 (Xp11.23) and FOXP4 (6p21.1) with similar 110 amino acid DNA-binding domain termed winged helix\forkhead domain [3], since 19 Fox gene subfamilies (A-S) were identified with 50 genes in humans so far [4]. FOXP proteins play important roles in the regulation of gene transcription in associated with immune function [4], carcinogenesis [5, 6], differentiation [7, 8] and angiogenesis [6, 9, 10].

Accumulating evidence reveals that FOXP1 regulates development of B cells [11], FOXP2 controls language development [12] and FOXP4 mediates development of T cell [13]. Furthermore, FOXP3, so called scurfin, is critically involved in differentiation and function of regulatory T cells or CD4+/CD25+ regulatory T (Treg) cells for cancer immunotherapy [14, 15]. It is well documented that FOXP3 modulates Treg development and functions [16] by immune evasion of tumor cells through imbalance of immunoediting and immunosurveillance in some cancers [17].

Additionally, it is well documented that FOXP proteins interact with other molecules and signaling pathways. FOXP1 has protein-protein interaction with NFAT1 to enhance breast cancer cell motility [18] and FOXP2 is essential in growth arrest of 143 osteosarcoma cells via p21 activation [19]. Also, FOXP3 is associated with IL-17 [20], RUNX1 [21], STAT3 [22], NF-κB [23], FOXO3 [24] and other cofactors such as EOS (*Ikzf4*) [25], interferon regulatory factor 4 (IRF4) [26], special AT-rich sequence-binding protein-1 (SATB1) [25, 27] and GATA1, while FOXP4 is closely associated with miR 4316 in breast cancer cells [28], miR 491–5p in osteosarcoma [29] and miR 338-3p in hepatocellular carcinoma [30].

Nevertheless, the underlying functions of FOXP proteins in cancer progression and immunology still remain unclear and confused to readers. Thus, the present review highlighted research history, dual functions of FOXP family as a tumor suppressor or an oncogene, their interaction with other proteins and noncoding RNAs, and cellular therapy targeting FOXP proteins in cancer progression, their clinical implications and finally suggested research perspectives.

## Overview of FOXP family: structure and domains and research history

**Fig. 1:**
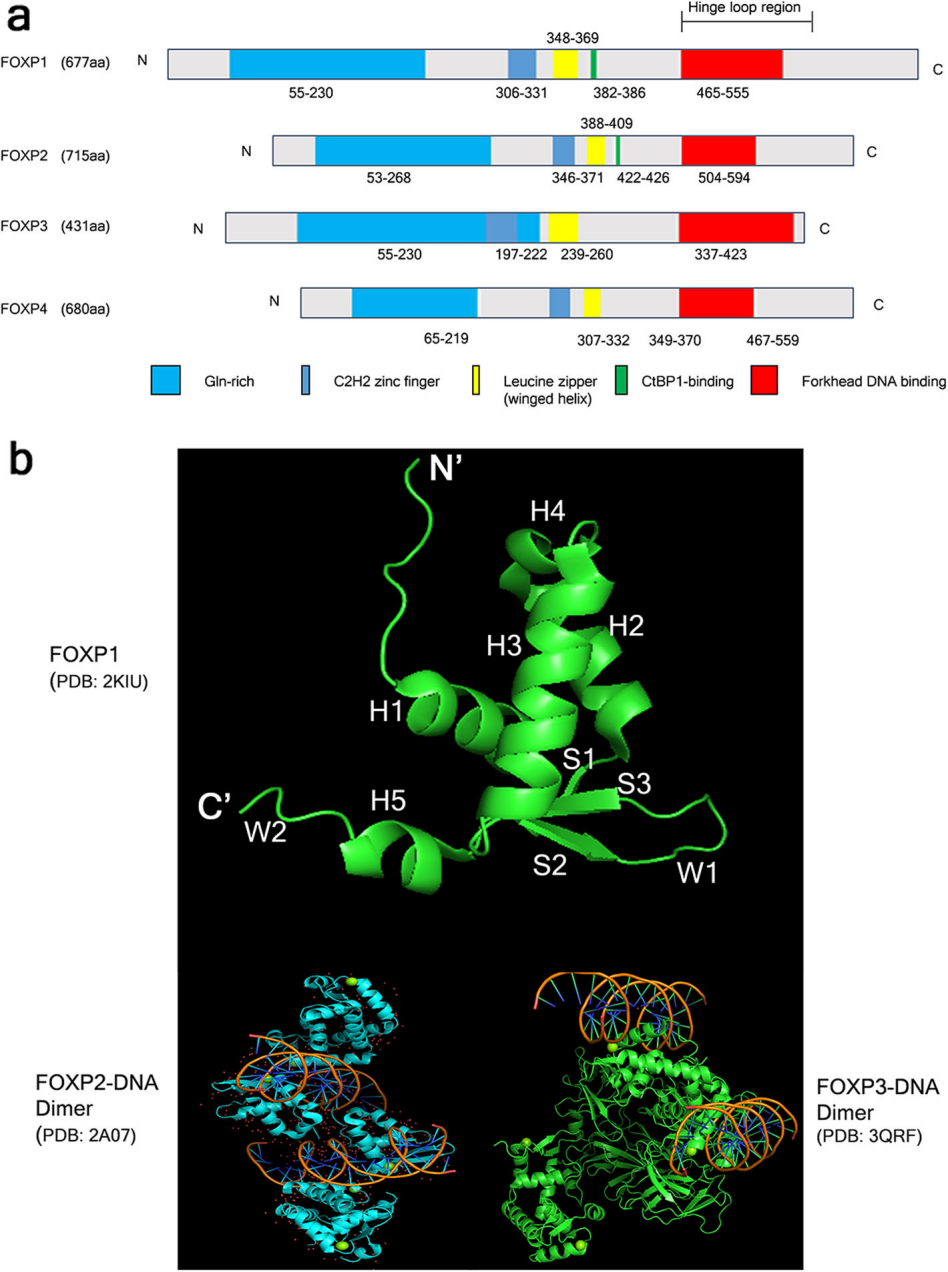
Domains and crystal structures of FOXP family. **a** Domains of FOXP1, FOXP2, FOXP3 and FOXP4. FOXP members share a highly conserved C2H2 zinc finger domain, leucine zipper domain, Forkhead DNA binding domain and about 50 residues N terminal domain. Also, FOXP1 and FOXP2 contain CtBP1 binding domain different from FOXP3 and FOXP4. **b** Crystal structures of FOXP1 (PDB: 2KIU), FOXP2-DNA complex (PDB: 2A07) and FOXP3-DNA complex (PDB:3QRF) by using The PyMOL Molecular Graphics System Version 2.3.0

FOXP (Forkhead box P) family proteins share a highly conserved C2H2 zinc finger domain, leucine zipper (winged helix) domain (WHD) consisting of β-sheets, α-helices and wings or loops like a helix-turn helix-like motif [31], winged helix Forkhead DNA binding domain (FHD) and about 50 residues N terminal domain [32] (Fig. 1a). Of note, FOXP1 and FOXP2 contain C-terminal-binding protein 1 (CtBP1) binding domain different from FOXP3 and FOXP4 [33–36]. Also, tertiary structure of FOXP1 forkhead domain contains five α-helices (H1–5), three β-strands (S1, S2 and S3) and two wings (W1 and W2) [37, 38] and also FOXP2 and FOXP3 have similar crystal structure of FOXP1 with their specific dimers (Fig. 1b). Though the FHDs of FOXP1, FOXP2, FOXP3 and FOXP4 all have a C-terminal winged helix FHD to be dimerised, FOXP3 dimer is considered more stable than FOXP2 or FOXP1 dimer [36]. Notably, Mendoza et al. [39] identified homo- hetero dimers and an oligo composed of FOXP1/2/4 complex in HEK 293 cells and brain. Additionally, of two independent subdomains required for transcriptional repression activity, subdomain 1 with a highly conserved leucine zipper similar to that of N-Myc gives homo- and hetero-dimerisation to FOXP1/2/4 proteins. In contrast, subdomain 2 with a binding motif for the corepressor protein C-terminal binding protein 1 (CtBP-1) is found only in FOXP1 and FOXP2 but not in FOXP4,while FOXP3 binds to RUNX to repress target gene expression [13, 40].

**Fig. 2:**
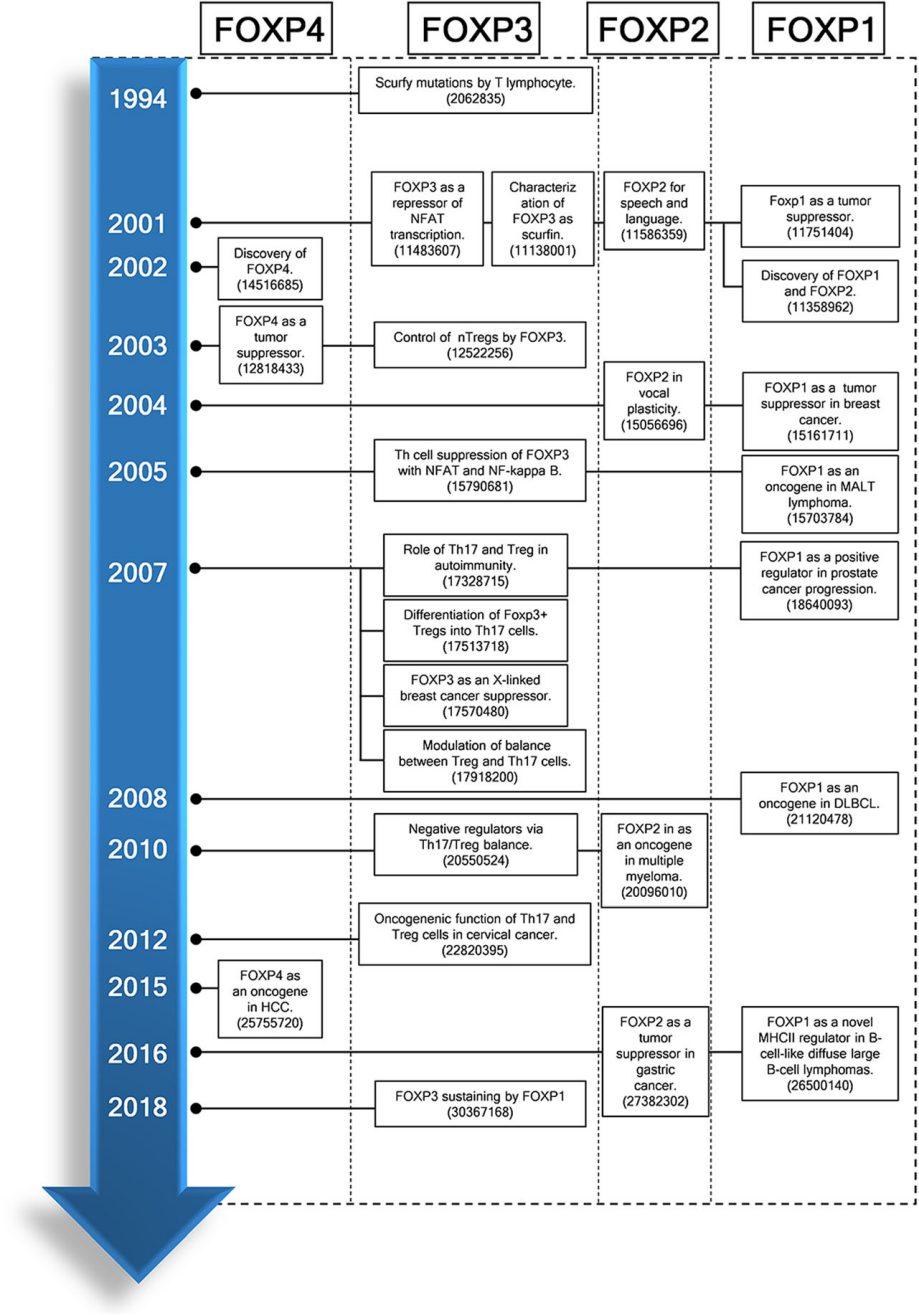
Timeline for FOXP family research history. DLBCL, diffuse large B-cell lymphoma; FOXP1–4, forkhead box P1–4; NFAT, Nuclear factor of activated T-cells; Th17, T helper 17; Treg, T regulatory; nTreg cells, naïve T regulatory cells; NF-kappa B, Nuclear factor kappa B. The numbers in references indicate PMID of PubMed

Looking back on research history of FOXP family, as first discovery of FOXP family, Godfrey et al. [41] suggested that T lymphocytes mediate scurfy lesions in abnormal thymic environment in 1991, since regulatory T cell-deficient scurfy mice induce severe autoimmune disorders, leading to death (Fig. 2). Thereafter, Brunkow et al. [42] first coined FOXP3 for scurfin essential for normal immune homeostasis, and human Treg cells were further characterized as CD4^+^CD25^+^ T cells by Taams and his colleagues [43] in 2001, since Sakaguchi et al. [44] reported that CD4^+^CD25^+^ cells enhance self-tolerance by immunosuppression in 1995. Then Shu and his colleagues [45] identified and characterized FOXP1 and FOXP2 in the lungs of mice. Also, Banham et al. [46] suggested FOXP1 as a novel tumor suppressor candidate localized to the chromosome 3p 14.1 region. However, FOXP1 was found as a tumor suppressor in breast cancer [47] and as an oncogene in MALT lymphoma [48]. Additionally, FOXP2 was recognized as an oncogene in several lymphomas including multiple myeloma, and as a tumor suppressor in gastric cancer [49] and hepatocellular carcinoma (HCC) [50] since Lai et al. [51] first demonstrated that FOXP2 is critically involved in a severe language and speech disorder in 2001.

Hori et al. [52] for the first time identified that FOXP3 is a key regulatory gene for the development of Treg cells in 2003 and Schubert et al. [53] reported that scurfin or FOXP3 represses NFAT transcription factor and cytokine production and proliferation by CD4^+^ T cell activation. Next, Teufel et al. [13] demonstrated FOXP4 as a tumor suppressor in patients with kidney tumors in 2003 and Wang et al. [30] reported FOXP4 as an oncogene in HCC in 2015. Consistently, FOXP4 depletion inhibits proliferation of HCC as a negative regulator of miR-338-3p [30]. Of note, the important role of Th17/Treg ratio has been a hot issue in cancers [54] and autoimmune diseases [55, 56], since Treg cells can be differentiated into Th17 cells [57] and then the significance of Th17/Treg from Th1/Th2 was revealed in immune response [58]. From this research chronicle, extensive research has been conducted targeting FOXP family.

## Dual biologic functions of FOXP family proteins as a tumor suppressor or an oncogene

Despite accumulating evidence on dual functions of FOXP proteins as an oncogene or a tumor suppressor in specific cancer types [59, 60] and their related signaling pathways, it still remains unclear under what factors or circumstances the FOXP proteins act a tumor suppressive or oncogenic role. It is well documented that FOXP1 is overexpressed with poor prognosis in diffuse large B-cell lymphoma (DLBCL) [61–64], primary cutaneous large B-cell lymphomas (PCLBCL) [65, 66], follicular lymphoma [67] and gastric mucosa-associated lymphoid tissue lymphoma (MALT) [68] as an oncogene. Consistently, Wang et al. [69] reported that FOXP1 depletion reduced the proliferation of hepatocellular carcinoma via G1/S phase arrest and decreased phosphorylation of retinoblastoma protein (Rb). Notably, Brown et al. [70] indicated that the growth of DLBCL is mediated by suppression of MHC class II expression and immune response signatures and activation of Wnt/β-catenin signaling induced by FOXP1 [71]. Also, Bates et al. [47] reported that nuclear FOXP1 is significantly co-expressed with estrogen receptor beta or alpha in ER positive MCF-7 breast cancer patients following tamoxifen treatment, though they do not directly affect each other by siRNA transfection [72]. Also, FOXP1 works as an oncogene by activating chromosome translocations under the control of immunoglobulin heavy chain (IGH) enhancers [60, 73].

In contrast, FOXP1 is also known as a tumor suppressor, since FOXP1 gene maps to a tumor suppressor locus at 3p14.1 and so loss of FOXP1 expression is associated with a poor outcome in in breast cancer [74]. Furthermore, overexpression of FOXP1 inhibits proliferation and invasion in U251 glioma cells [75], while knockdown of FOXP1 promotes the development of lung carcinoma [76]. Similarly, FOXP1 represses AR-induced transcriptional activity or histone modification as a tumor suppressor [77, 78]. Interestingly, previous evidence reveals that FOXP1 and FOXP2 exert functional cooperativity during development. Indeed, *FOXP2*^*−/−*^*FOXP1*^*−/+*^ mice showed severe developmental defects and perinatal lethality compared to *FOXP2*^*−/−*^*FOXP1*^*+/+*^ mice [5]. Furthermore, FOXP1 is also known to interact with FOXP3 through NFAT-IL-2 promoter DNA complexes [74, 79].

Recently, critical roles of FOXP2 have been demonstrated in cancer progression as a tumor suppressor, though FOXP2 mutations are well known to cause language and speech development deficits. Also, FOXP2 was reported to suppress the transcriptional activity of target genes through the Zinc finger domain and also binds to domain for C-Terminal Binding Protein-1 (CtBP1) for suppressing E-cadherin and promoting invasion [59]. Furthermore, Cuiffo et al. reported that downregulation of FOXP2 enhances tumor initiation in breast cancers as a putative tumor/metastasis suppressor [80]. Also, FOXP2 was downregulated in hepatocellular carcinoma (HCC) tumor tissues with poor overall survival rate and its downregulation significantly promoted the invasiveness of HCC [50]. In addition, FOXP2 is essential for regulation of p21 in 143B osteosarcoma cell growth inhibition [19]. Of note, Morris et al. claimed that phosphorylation at Ser557 is identified as another means of regulating the transcriptional functions of FOXP2 [81]. Furthermore, FOXP2 is regarded as a SUMO target protein at cellular level, since FOXP2 is covalently modulated by both SUMO1 and SUMO3. SUMOylation of FOXP2 is significantly disturbed by a specific SUMO Specific Protease 2 (SENP2), since SUMOylation modulates transcriptional activity of FOXP2 in targeting downstream target genes (DISC1, SRPX2, and MiR200c) by reporter gene assay [82].

In contrast, mutations of transcription factor FOXP2 were shown in neoplastic plasma cells [83] and overexpression of FOXP2 is associated with high risk of early PSA recurrence in erythroblast transformation-specific-related gene (ERG) fusion-negative prostate cancers [84].

FOXP3 promotes the immune evasion as Treg cell marker suppressing immune response against cancer, while FOXP3 at the Xp11.23 revealed good prognosis in breast cancers as a tumor suppressor [85–88] by regulating HER-2/ErbB2 [88] or SKP2 [89, 90] oncogene. Furthermore, it is noteworthy that FOXP3 functions as dual roles through interaction with other transcription factors nuclear factor kappa-B (NF-κB), nuclear factor of activated T cells (NFAT) [91], and acute myeloid leukemia 1 (AML-1) [92] in the tumor microenvironment.

FOXP4 is closely associated with FOXP1 and FOXP2 with 54 and 60% identity, respectively since FOXP4 forms a large multidomain transcriptional repressors with FOXP1 and FOXP2 [40], while FOXP3 and FOXP4 protein sequences are merely 47% identical in the aligned sequence region [13]. FOXP4 was overexpressed in A549 and H1703 non-small cell lung cancer (NSCLC) cells and conversely FOXP4 depletion markedly reduced the growth and invasion of above two NSCLCs [93]. Furthermore, FOXP4 gene was closely associated with prostate cancer risk in Chinese men [94, 95] and also long non-coding RNA FOXP4-AS1 is suggested a poor prognostic factor in colorectal cancer [96] and osteosarcoma [97]. In contrast, FOXP4 was significantly downregulated in patients with kidney cancers [13]. Overall, despite accumulating evidence on dual functions of FOXPs, further study is required to verify the dual role mechanisms of FOXP proteins in association with their related molecules under specific microenvironment or phosphorylation condition in the near future.

## Regulating tumor progression by FOXP3 in the tumor microenviroment

It is well documented that FOXP3 is a key transcription factor for development and function of Treg cells [98]. Treg cells are produced from the thymus, and the periphery, by constitutively expressing glucocorticoid-induced TNF receptor family-related gene (GITR), cytotoxic T lymphocyte associated antigen 4 (CTLA-4) and IL-2 receptor (IL-2R) α chain (CD25) [99, 100]. Treg cells induce immunosuppression by CTLA-4–mediated downregulation of costimulatory molecules or IL-2 deprivation on antigen-presenting cells (APCs), and by secretion of cytokines, such as IL-10 or TGF-β. Thus, Treg cells suppress tumor-specific CD8^+^ T cell cytotoxicity through TGF-β signaling [101] and some molecules including nuclear factor of activated T cells (NFAT) [15] and Runt-related transcription factor 1 (RUNX1) [92] are found to bind to the promoter regions of FOXP3-regulated genes for activation of Treg cells. FOXP3 overexpression of Tregs may promote tumor cell growth in non-small cell lung cancer (NSCLC) microenvironment [102].

FOXP3 regulates immune system as a specific marker for CD4^+^/ CD25^+^ or CD4^+^/CD25^−^ Treg cell development and function [17, 103, 104]. CD4^+^/CD25^+^/FOXP3^+^ Treg contributes to immunosuppression and cancer progression by reducing the anticancer immunity of CD4^+^ or CD8^+^ effector T cells [17, 105]. Two major populations of Treg cells have been defined as peripherally induced Treg (iTreg) cells and thymically derived natural Treg (nTreg) cells. CD4^+^CD25^+^FOXP3^+^ nTreg cells derived from thymus are known to modulate immune disorders such as autoimmunity, allergy, and graft rejection by suppressing activation of naïve T cells, effector T cells and memory CD4^+^ and CD8^+^ T cells [106]. iTreg cells, so called as type 1 regulatory T cells (Tr1), are developed from naïve T cells in the periphery during an active immune response including antigens, IL-2, IL-10 and TGF-β [107]. Furthermore, human FOXP3 expressing nTreg cells can be subdivided into CD25^+^/CD45RA^+^/FOXP3^lo^ (resting Treg cells; rTreg cells), CD25^hi/^CD45RA−/FOXP3^hi^ (activated Treg cells; aTreg cells), and CD25^+^/CD45RA^−^/FOXP3^lo^ (non-Treg cells) [108, 109]. Interestingly, Whiteside [110] suggested that iTreg cells should be depleted and nTreg cells are promoted in cancer patients, since iTreg cells produce immunosuppressive cytokines, notably TGF-β as well as prostaglandin E2 resistant to oncological therapy, while FOXP3^+^ nTreg cells are responsible for peripheral tolerance to avoid autoimmune disease [111].

In addition, CD4^+^CD25^+^ regulatory T cell deficiency due to loss-of-function mutations of FOXP3 gene induces the lethal autoimmune syndromes observed in FOXP3-null mice or FOXP3-mutant scurfy mice [98]. Consistently, the infiltration of effector Treg cells into tumor cells indicates poor prognosis of overall survival (OS) [87, 112] and FOXP3 is overexpressed in pancreatic [113], prostate [114] and gastric [115] cancers by suppressing antitumor immunity [116] and inducing effector CD4^+^ T cell death by activation of proapoptotic protein Bad and Bim [101].

## The interplay between FOXP family members and other molecules

### FOXP1 and interleukins (IL-7 and 21)

FOXP1 works as a negative regulator of tumor-specific CD4+ T helper 7 (Th7) cells for cancer immunotherapy, since mature naïve CD4^+^ T cells proliferate to exert antitumor effect programmed by IL-7 only in the absence of FOXP1 [117, 118]. Similarly, CD8^+^ T cells lacking the transcription factor FOXP1 show function and effector phenotype of IL-7, since FOXP1 represses expression of IL-7 receptor α-chain (IL-7Rα), phosphorylation of MEK and ERK [119]. Furthermore, FOXP1 suppresses the antitumor function of interleukin 21 (IL-21) to stimulate the secretion of IFNγ from CD4^+^ T or CD8^+^ T cells in estrogen positive MCF-7 breast cancers [70, 120]. In contrast, De Silva et al. indicated that FOXP1^hi^ supernatant reduced lymphocyte migration by secretion of chemokines such as CXCL9, CXCL10, CXCL11, CXCL13, CX3CL, CCL20, IL-7, IL-21, and IFNγ compared to FOXP1^lo^ supernatant [121]. Nevertheless, the mechanism by which FOXP1 represses IL-7 or IL-21 still remains unclear to date.

### FOXPs and nuclear factor of activated T cell (NFAT)

Nuclear factor of activated T cells (NFAT) is an inducible nuclear factor binding to the antigen receptor response element-2 (ARRE-2) of IL-2 promoter in human T cells and also is involved in cell proliferation, survival, invasion, migration and angiogenesis [122]. NFAT family include five members such as four calcium-responsive isoforms named NFAT1 (NFATp or NFATc2), NFAT2 (NFATc or NFATc1), NFAT3 (NFATc4), NFAT4 (NFATx or NFATc3) and a tonicity-responsive enhancer-binding protein (NFAT5 or TonEBP) [122, 123]. The NFAT isoforms are constitutively activated in several cancer types [122, 124]. Interestingly, FOXP proteins form cooperative complexes with NFAT [74] and so the crystal structures of the FOXP2–NFAT2 DNA complex are also conserved with FOXP1 and FOXP3 [15, 123]. Also, NFAT1 depletion inhibited invasion and migration of human non-small cell lung cancer [125] and NFAT overexpression promoted invasion in breast cancers via upregulation of cyclooxygenase-2, α6β4 integrin and glypican-6 [126–128]. Likewise, Oskay et al showed that FOXP1 directly binds to NFAT1 on DNA and promotes migration in MDA-MB231 breast cancer cells [18]. However, FOXP1 binds poorly to the ARRE2 composite site in the absence of NFAT1 [15]. Overall, NFAT1 closely interacts with FOXP1 or FOXP3 in cancer progression.

### FOXPs and p53/p21

It is well documented that p53 suppresses tumorigenesis by regulating apoptosis, metabolic networks, free radical and senescence [129]. Recently, Jung et al. demonstrated that p53 induction by genotoxic reagent upregulates FOXP3 expression and conversely FOXP3 is regulated in a p53-dependent manner by MDM2 inhibitor Nutlin-3 [130]. Of note, FOXP3 induced cellular senescence in MCF7 and HCT116 cells via activation of p53/p21 and reactive oxygen species(ROS) production [131]. Furthermore, FOXP1 known as a B cell oncogene is reduced by miR-34a via p53 networks [132], indicating .the cloe interaction between FOXP1/3 and p53 signaling.

Accumulating evidence demonstrate that the cyclin-dependent kinase inhibitor *p21* ^*WAF1/CIP1*^ is a widely-characterized p53 target gene during cell cycle arrest [133, 134]. Of note, Gascoyne et al. indicated that FOXP2 activation preceded up-regulation of p21^WAF1/CIP1^ in 143B osteosarcoma cells [19]. Likewise, FOXP2 overexpression upregulated the expression of p21 in hematopoietic stem cells (HSCs). However, though p21 is known a downstream effector of gp130/STAT3 activation [135], exogenous STAT3 promoter IL-6 could not rescue reduction of *p21*^*WAF1/CIP1*^ expression following FOXP2 depletion, implying that FOXP2-dependent regulation of *p21* ^*WAF1/CIP1*^ independent of IL-6 [19]. Also, it was known that FOXP2 regulates p21 independent of p53 status in cell lines (143B mutant, MG-63 null, U2OS wild-type, SAOS-2 null) different from FOXP1 [136, 137] proteins, which should be further investigated in specific cell lines and in vivo.

### FOXP3 and Interleukin-17

Emerging evidence shows that Treg population is observed in tumor infiltrating lymphocytes of several cancers such as breast cancer [138], gastric cancer [139], pancreatic cancer [140] and, colorectal cancer [141] and lung cancer [142]. Interestingly, CD4 + CD25 + FOXP3 + (GFP+) T cells can differentiate into T helper 17 (Th17) cells in the presence of IL-6 [57] and Th17 cells, one of the CD4+ T cells, can produce IL-17 to protect cells against microbial infection [143]. In contrast, activation of Treg cells reduces antipathogenic or anticancer immunity, leading to cancer progression and infection [144].

Hence, the balance between FOXP3+ Treg cells and Th17 cells is considered an important factor for treatment of autoimmune diseases [145] and cancers [146]. Indeed, Maruyama et al. [54] reported that the infiltration of Th17 cells gradually decreased compared to increased Treg cells in gastric cancer progression. However, Hou et al. [20] claimed that Th17 cells and FOXP3-expressing T cells were significantly increased in uterine cervical cancer and cervical intraepithelial neoplasia while the ratio of Th17/FOXP3 Treg cells was decreased in tumor-infiltrating lymphocytes (TILs). Consistently, Beyer [147] suggested a novel strategy to suppress expansion and differentiation of naïve Treg cells induced by IL-2 therapy, since Treg cells induce immunosuppression in neoplastic patients. For reprogamming Treg cells into Th17 like cells, Sharma et al. [148] suggested that indoleamine 2,3-dioxygenase (IDO) inhibitor and antitumor vaccine converted Treg cells into Th17 phenotype cells in B16 melanoma mouse model. Similarly, Bahan et al. [149] reported that high dose of oligonucleotides (CpG) treatment directly reprogrammed splenic FOXP3 Treg cells to express IL-17 with efficiency of 6–7% in IDO-KO mice model.

In contrast, Xu et al. [57] suggested that activated FOXP3^+^ Treg cells have potential to stimulate CD4^+^CD25^−^FOXP3^−^T cells or can differentiate into Th17 phenotype cells in the presence of IL-6 regardless of exogenous TGF-β. Thereafter, the property of IL-17^+^FOXP3^+^ T cells has been recognized with low expression of Helios [150] and overexpression of ICOS [139] and RORγt for distinguishing peripherally induced and thymic-derived FOXP+ Treg cells. Thus, IL-17^+^FOXP3^+^ T cells were considered as an intermediate differentiation stage between Th17 cells and Treg cells, since Treg cells can be converted into IL-17^+^FOXP3^+^ T cells by stimulation of TGF-β and/or IL-6 [151]. Accumulating evidence reveals that IL-17 + FOXP3+ T cells are shown highly in colon [152] and esophageal [153] cancers, inflammatory bowel disease [154], periodontitis [155], and rheumatoid arthritis [156]. Nevertheless, further studies are required to explore the mechanisms and interplay with other molecules in the differentiation of IL-17^+^FOXP3^+^ T cells, and to assess clinical implications targeting the balance between FOXP3^+^ Treg cells and Th17 cells in the near future.

### FOXP3 and RUNX

RUNX proteins such as RUNX1 (AML1) and RUNX3 induced by TGF-β play a critical role in embryonic development, hematopoiesis and T cell development by regulating CD4, CD8 and lymphokine genes [157, 158]. Emerging evidence indicates that the complex of core-binding factor subunit beta (CBFβ) and runt-related transcription factor 1 and 3 (RUNX1 and 3) is essential for Treg suppressive function [7, 159]. Recouvreux et al. suggested that RUNX1 plays a critical role in breast tumor progression, only depending on FOXP3 availability [21]. Also, *RUNX1* is known as an important target for chromosomal translocations of leukemias with CD4^+^CD25^high^FOXP3^+^ Treg cells [92, 160], while *RUNX3* methylation and silencing are observed in several epithelial cancers [161, 162]. Also, the role of RUNX1 or RUNX3 should be explored in the differentiation of I IL-17^+^FOXP3^+^ T cells in association with mTOR, CNOT2, HIF-1α and RIF-4 that are involved in tumor progression and immune tolerance.

### FOXP3 and STAT3/5

There is accumulating evidence that STAT3 is involved in cancer initiation and development as an oncogenic transcription factor [163]. Regarding relationship between FOXP3 and STAT3, Hossain et al. revealed that FOXP3 silencing decreased the expression of STAT3-related genes such as *IL-6, VEGFA, C-Myc, BCL2L1*, and *CCND1,* but not *TGF-β1 in tumor induced regulatory T cells by qRT-PCR analysis* [22]. Conversely, STAT3 promoter IL-6 induced DNA-methyltransferase 1 (DNMT1) expression and promoted STAT3-dependent methylation of FOXP3 in Treg cells [164]. Also, Lam et al. demonstrated the important role of Cdk5 in phosphorylation of STAT3 (S727) to bind to FOXP3 gene in CD4^+^ T cells [165].

Additionally, activated STAT5 is associated with suppression of antitumor immunity, since STAT5 plays a critical role in the function and development of Treg cells to promote proliferation, invasion, and survival of tumor cells [166]. Furthermore, **s**everal studies demonstrated that STAT5 mediates the critical link between the IL-2/15 and FOXP3. Thus, in T-cell-specific STAT5-null mice, CD25- and FOXP3-expressing cells were reduced and also STAT5 was detected to bind to gamma interferon-activated sequence (GAS) sites of FOXP3 promoter [167, 168]. Similarly, Wang et al showed that activated STAT5 is associated with increased FOXP3 expression in melanoma cells and lymphocytes [169]. Hence, the pivotal role of STAT3 or STAT5 should be extensively explored in FOXP3^+^ Treg cells in vitro and in vivo.

### FOXP1/3 and FOXO3a/1

Among FOXO subfamilies, FOXO3a is well known to act as a tumor suppressor by inducing apoptosis and cell cycle arrest [170]. Interestingly, FOXO3a phosphorylation as a downstream of E3 ubiquitin-protein ligase CBL-B increased FOXP3 expression in CBL-B deficient T cells and conversely FOXO3a depletion impaired TGF- β driven FOXP3 induction [171], since FOXO3a directly binds to the FOXP3 promoter in iTreg cells [172]. Furthermore, corporation of FOXO1 and STAT5 suppresses FOXP3 expression and production in response to the TCR signaling [173], since miR-182 downregulates FOXO1 [174] and PTPN2 dephosphorylates STAT5 [175], leading to suppression of FOXP3. Also, Du et al. reported that Mst1/Mst2 kinases enhanced FOXO3-mediated FOXP3 expression by maintaining the stability of FOXO1/3 proteins through phosphorylation of S212 and S207 and inhibited TCR induced Akt activation [176], implying the role of Mst1/Mst2 kinases in FOXO3-mediated FOXP3 expression. In addition, Bi et al. [118] reported that FOXO1 acts as a negative regulator and FOXP1 and a negative regulator of CD4^+^ T helper 9 (T_H_9) cell differentiation and antitumor activity. Likewise, van Boxtel et al. demonstrated that FOXO1/3 activation in FOXP1 depleted cells enhanced cell death, indicating the opposing roles between FOXO1/3 and FOXP1 [177]. Hence, the underlying mechanism of FOXO1 or FOXO3 mediated FOXP1/3 expression should be further explored in vitro and in vivo in tumor progression and chemoresistance.

### FOXP3 and NF-κB

It is well documented that NF**-**κB is involved in inflammation, proliferation, cell adhesion and tumor progression [178]. Of note, Hao et al. revealed that FOXP3 suppresses cell migration by inhibition of NF-κB activity and COX-2 expression in gastric cancers [179]. However, the tumor suppressor role of FOXP3 was disturbed under inflammatory microenvironment in gastric cancers, since FOXP3 interacts with two key transcription factors such as nuclear factor of activated T cells (NFAT) and NF-κB [91, 180]. Consistently, Wang et al. suggested that silencing of FOXP3 promoted the proliferative, migratory and invasive properties of A549 cells by downregulation of ZO-1, upregulation of vimentin and phosphorylation of NF-κB at protein level. Likewise, FOXP3 downregulation attenuated the expression of LIM Domain Only 2 (LMO2) but increased the expression of an oncogenic transcription factor T-cell acute lymphocytic leukemia protein 1 (TAL1) at mRNA level in T-cell acute lymphoblastic leukemia (T-ALL) cells [181]. However, Chu et al. demonstrated that FOXP3 depletion downregulated cyclin D1 and NF-κB subunit p65, but upregulated caspase-3 levels in K1 and WRO thyroid cancer cells [182]. Also, Jia et al. indicated that blockade of toll-like receptor 4 (TLR4) signaling induced downregulation of FOXP3 after blocking NF-κB in A549 cells [183]. Here microenvironmental conditions where FOXP3 acts as a tumor suppressor or an oncogene should be clearly determined in specific cancers in association with TRL4, NFAT and NF-κB signaling in the future.

### FOXPs and VEGF

It is well documented that angiogenesis related molecules including VEGF play pivotal role in carcinogenesis [184]. Le et al. reported that FOXP3 suppresses VEGF signaling to exert anti-angiogenic or anti-metastatic effect in MDA-MB-231 breast cancer cells [185, 186]. However, Tang and his colleagues demonstrated that FOXP3 is positively correlated with VEGF-C in lymphangiogenesis of cervical cancer [187]. Likewise, FOXP1 promoted proliferation, migration and tube formation in cultured endothelial cells [9]. Also, Wan et al. demonstrated the correlation between FOXP1 and VEGF in the patients with renal carcinoma [188]. Furthermore, He et al. suggested that FOXP1 and FOXP2 upregulates levels of angiogenic factor such as VEGF with G patch and FHA domains 1 (AGGF1) [189] and induces angiogenesis in glioma cells [190]. Overall, it is demonstrated that FOXP proteins are closely associated with VEGF signaling.

### FOXPs and noncoding RNAs

Emerging evidence suggests that FOXP members modulate various noncoding RNAs during cancer development and progression, since noncoding RNAs (ncRNAs) are RNA molecules that are not translated into proteins, including transfer RNAs (tRNAs) and ribosomal RNAs (rRNAs), as well as small RNAs such as microRNAs (miRNAs), siRNAs, circular RNA and the long ncRNAs (lncRNAs) [191]. For instance, FOXP1 induction by inhibition of miR-9 promoted tumor growth, while FOXP1 knockdown suppressed the growth of epidermal growth factor receptor (EGFR) dependent cancers [192]. Also, downregulation of FOXP1 increased miR-34a level as a tumor suppressor in gastric diffuse large B-cell lymphoma (gDLBCL) cells [193]. Furthermore, elevation of miR-199a and repression of FOXP2 are prominent features of malignant breast cancer with poor survival rate [194]. Likewise, miR-181d-5p [195], miR-374b-5p [196], miR-122 [197], miR-150 [198] and miR-504 [199] act as a tumor suppressor by inhibition of FOXP1, while miR-376a [200] and miR-139 [201] work as a tumor suppressor via inhibition of FOXP2 in lymphoma and osteosarcoma. Additionally, miR-146 [23, 202], miR-7 and miR-155 [203] induced by FOXP3 act as a tumor suppressor in breast and prostate cancers. Notably, Liu et al. revealed that FOXP3-induced miR-146a/b suppressed tumor cell proliferation and enhanced apoptosis by inhibition of NF-κB activation through suppression of interleukin-1 receptor-associated kinase 1 (IRAK1) and TNF receptor associated factor-6 (TRAF-6) in MCF-7 breast cancer cells [202]. Likewise, McInnes et al. showed that FOXP3 induced miR-7 and miR-155 to target oncogenic SATB1 in BT549 breast cancer cells [203], whereas overexpression of miR-338-3p inhibited proliferation of hepatocellular carcinoma cells and induced cell cycle arrest partially through the downregulation of FOXP4 [30]. Of note, miR-338-3p [30], miR-491–5p [29] and miR-138 [93, 204] were downregulated in HCC, osteosarcoma and non-small lung cancer cells, targeting FOXP4. In addition, lncRNA MALAT1 [205] and SNHG12 [206] promoted proliferation of multiple myeloma and glioma targeting FOXP1, while lncRNA UFC1 [207] and 7SL [208] enhanced proliferation of cervical cancer and osteosarcoma, targeting FOXP3 and FOXP4, respectively. Notably, circular RNA SHKBP1 was upregulated in malignant glioma targeting FOXP1 or FOXP2 [190], while circular RNA ZNF609 [209] and MYO9B [28] were upregulated in renal cancer and breast cancer, respectively, targeting FOXP4. Taken together, the critical roles of these miRNAs, lncRNAs, circular RNAs should be further investigated during antitumor or oncogenic effects of FOXP families in vitro, in vivo and clinically in the future.

## Clinical application and cellular cancer immunotherapy

Among clinical trials Dr. Sylvain Ladoire performed clinical trial (ClinicalTrials.gov Identifier: NCT01513408) at Centre Georges Francois Leclerc with 500 participants with a topic, “Prospective study of the relevance of T lymphocytes tumor infiltrates D8 and FOXP3 as a new immune prognostic biomarker in breast cancer treated by neoadjuvant chemotherapy to verify that accumulation of regulatory T-lymphocytes expressing FOXP3 is associated with poor prognosis in breast cancer patients. Also, another clinical trial (ClinicalTrials.gov Identifier: NCT03718923) on FOXP1 related neurodevelopmental disorders is underway at The Seaver Autism Center for Research and Treatment, New York. Also, pilot study (ClinicalTrials.gov Identifier: NCT01538485) was completed to assess the effects of Vitamin D supplementation on the number of regulatory FOXP3^+^ T cells in the gastrointestinal mucosa in healthy women and men in Austria in 2012. Besides, graft rejection studies in renal transplant recipients (ClinicalTrials.gov Identifier: NCT01446484) and liver transplant recipients (ClinicalTrials.gov Identifier: NCT01678937) were conducted targeting FOXP3. Here it was found that clinical trials have been conducted targeting FOXP1 or FOXP3. Emerging evidence indicates that FOXP3 regulates Treg development and functions [16] to induce the immune evasion of tumor cells through imbalance of immunoediting and immunosurveillance [17] and. FOXP1 acts as a transcriptional regulator for primary human CD4^+^ T cells [210]. Recently dendritic cells (DCs) based immunotherapy has been on the spotlight for cancer therapy, since DCs are considered the most powerful antigen-presenting cells (APCs) activating naïve and memory immune responses [211]. Through a lot of clinical trials with DCs via various routes (intradermal, intranodal, intravenous, subcutaneous, intratumoral) [212], cancer immunotherapy efficacy by DC vaccine was limited mainly due to inhibition of immune response by tumor-secreted TGF-β and FOXP3 related Treg cells and low quality of DC production [213]. Notably, CD4^+^CD25^+^FOXP3^+^ (GFP^+^) T cells can differentiate into Th17 cells in the presence of IL-6 [57] and T helper 17 (Th17) cells, one of the CD4^+^ T cells, can produce IL-17 to protect against microbial infection [214], while excessive activation of Treg cells suppresses antipathogenic or anticancer immunity, leading to chronic infection and tumor progression [144]. The balance between FOXP3^+^ Treg cells and Th17 cells is considered an important target for treatment of autoimmune diseases [145] and cancers [146]. Thus, next generation DC immunotherapy is required for more effective cancer therapy. Thus, Treg depletion and Th17 booster can be a potent strategy for DC cancer immunotherapy and DC vaccines were suggested as a next generation cancer immunotherapy [215], only if the standardization and quality of DC vaccines can be upgraded to enhance migrating activity to the lymph nodes, presenting antigen and costimulation to T cells, and surviving long enough for optimal T-cell activation. In the same line, combination therapy of DCs with checkpoint inhibitors including ipilimumab [216] or other immune cells or oncolytic virus [216] is considered attractive in cancer therapy [216, 217]. In this regard, we suggest the cocktail of specialized DC vaccines and Th17 cells by reprogramming Treg cells into Th17 cells [146] or ex vivo expansion of Th17 cells from human PBMCs [218] is suggested as a next generation cellular cancer immunotherapy, which should be further investigated in vivo and clinically.

## Conclusions and perspectives

FOXP family consisting of FOXP1, FOXP2, FOXP3 and FOXP4 are involved in the embryonic development, immune disorders and cancer progression. Accumulating evidence reveals that FOXP family act as a tumor suppressor or an oncogene in several cancers. FOXP1 was overexpressed with poor prognosis in DLBCL, MALT, primary cutaneous large B-cell lymphomas and follicular lymphoma as an oncogene, while FOXP1 worked in breast, lung carcinoma and U251 glioma cells as a tumor suppressor. Also, FOXP2 was activated for up-regulation of p21 in 143B osteosarcoma cells, while FOXP4 was overexpressed in A549 and H1703 NSCLC cells along with prostate cancer risk. Likewise, CD4^+^/CD25^+^/FOXP3^+^Treg cells are overexpressed in pancreatic, prostate and gastric cancers through immunosuppression and cancer progression as an oncogene, while FOXP3 overexpression indicates good prognosis in patients with breast cancers as a tumor suppressor. Given that transcriptional activity of FOXP1, FOXP2, and FOXP4 is modulated by tissue-specific homo- and heterodimerisation via a zinc finger and a leucine zipper motif [33], their functional similarity is expected and so their more detailed protein-protein interactions and the molecular conditions for their dual roles as an oncogene or a tumor suppressor should be clarified in specific cancer types in the future, considering reports that dual functions of FOXP family may be closely associated with tumor microenvironmental factors such as dendritic cells (DCs), inflammatory cytokines especially in colon and esophageal cancers related to inflammation.

**Fig. 3:**
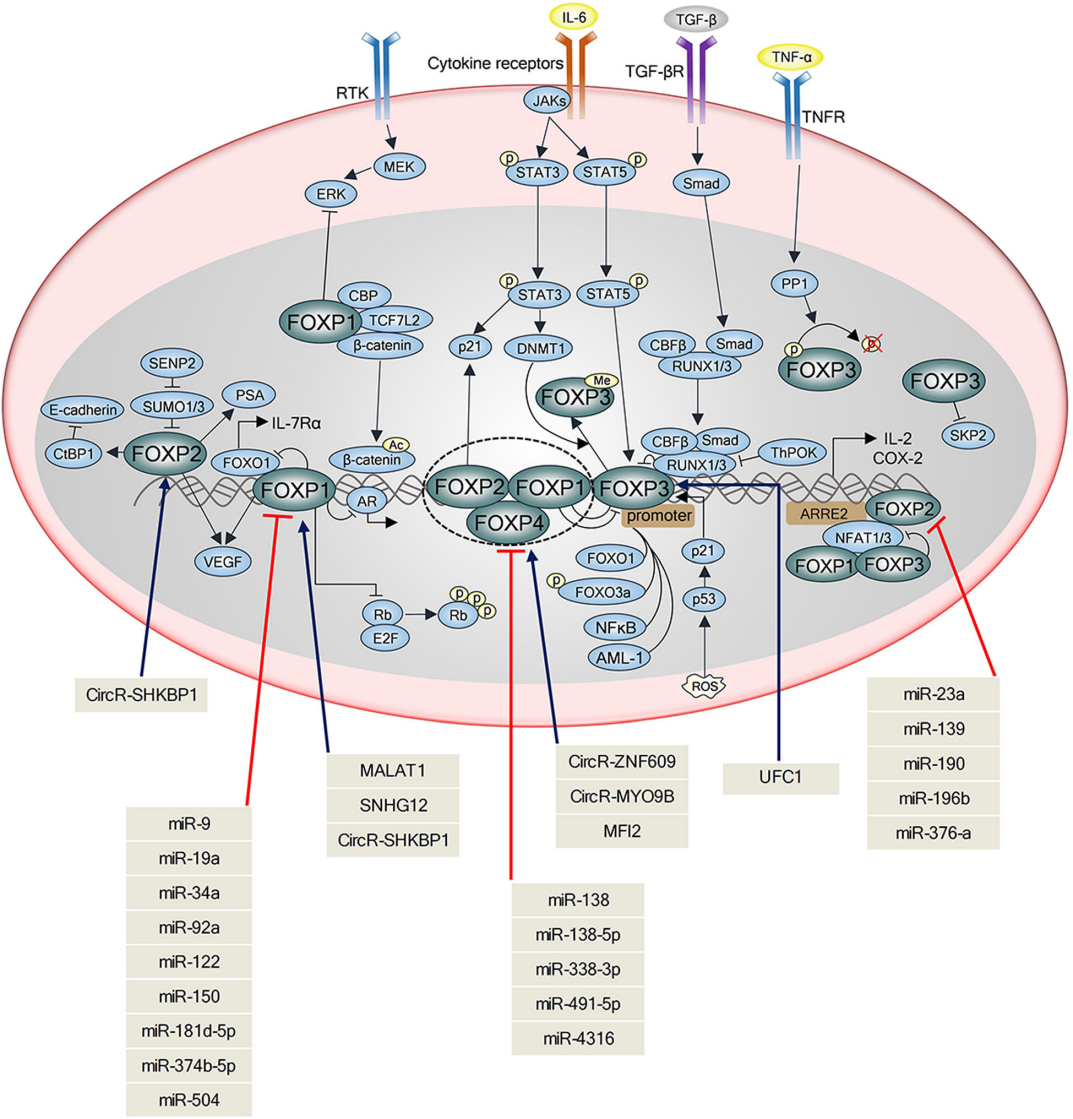
Interplay between FOXP family and their related molecules targeted by noncoding RNAs. FOXP family members consist of FOXP1, FOXP2, FOXP3 and FOXP4 that communicate with other molecules. Interleukin-6 (IL-6) activates the Janus tyrosine kinase (JAK) family members (JAK1, JAK2, and TYK2), leading to the activation of transcription factors of the signal transducer and activator of transcription (STAT) family including STAT3 and STAT5. Also, IL-6 induces DNA-methyltransferase 1 (DNMT1) expression and promotes STAT3-dependent methylation of FOXP3. FOXP2 overexpression upregulates the expression of p53/ p21, a downstream effector of gp130/STAT3. Transforming growth factor-β (TGF-β) activates phosphorylation of SMAD, which forms complex with CBFβ/RUNX1/3 for maintenance of *FOXP3*, but ThPOK blocks RUNX [219]. TNF-α stimulates protein phosphatase 1 (PP1) for dephosphorylation of FOXP3 (S418). FOXP3 interacts with two key transcription factors such as nuclear factor of activated T cells (NFAT) and NF-κB. FOXO3a phosphorylation increases FOXP3 and FOXO1 acts as a negative regulator and FOXP1. The receptor tyrosine kinases (RTKs) activate MEK-ERK signaling axis, which is repressed by FOXP1. Among noncoding RNAs, MALAT1, SNHG12 and CircRNA SHKBP1 activate FOXP1, while miR-9, miR-19a, miR-34a, miR-92a, miR-122, miR-150, miR-181–5p, miR-374-5p and miR-504 downregulate FOXP1. CircRNA SHKBP1 increases FOXP2, while miR-23a, miR-139, miR-190, miR-196b and miR-376a suppress FOXP2. UFC1 activates FOXP3 and miR-138, miR-338-3p and miR-491–5p downregulate FOXP4, while circR-SHKBP1, CircR-MYO9B and MFI2 upregulate FOXP4

Regarding interplay of FOXP members with their related molecules, FOXP1 is closely associated with IL-7, IL-21, NFAT, while FOXP2 is more related to p21 and FOXP3 is critically associated with IL-17, RUNX, STAT3/5, FOXO3a/1 and NF-κB. Also, it is well documented that FOXP members are regulated by miRNAs, lncRNAs, circular RNAs (Table 1, Fig. 3). Nonetheless, their detailed interactions are not fully understood as a tumor suppressor or an oncogene, which indicates further mechanistic study in vitro and in transgenic mouse model. Also, the underlying mechanisms that FOXO1/3 suppresses FOXP1/3 should be further examined in vitro and KO mice model.

**Table 1:** Effect of miRNAs, LNCRNAs and Circular RNAs on molecular mechanisms of FOXP family proteins and their related genes in several cancers

	Expression Level	Related gene	Function	Molecular mechanism	cancer	cell line	References
Tumor Suppressor miRNAs							
microRNA-181d-5p	down	FOXP1↓	cell proliferation↓ metastasis↓ EMT↓	miR-181d-5p-FOXP1 feedback loop	Osteosarcoma	MG63, SaOS2 U2OS, HOS	1
microRNA-374b-5p	down	FOXP1 ↓	cell proliferation↓ migration↓ EMT↓ cisplatin sensitivity↑	miR-374b-5p-FOXP1 feedback loop	Ovarian	SKOV3, 3AO A2780, OVCAR3	2
microRNA-122	down	FOXP1 ↓	apoptosis ↑	crosstalk	HCC	HepG2	3
microRNA-150	down	FOXP1 ↓	cell proliferation ↓	Myc↑ → miR-150↓ → Foxp1↑	trnasformation of FL to DLBCL	patient sample	4
microRNA-504	down	FOXP1 ↓	cell proliferation↓ cell cycle arrest↑ apoptosis↑	FOXP1 as a direct target of miR-504	Gliomas	U87, U373, U251,T98G, LN18, LN229, SF295	5
microRNA-9	down	FOXP1 ↓	tumorgenity	ΔEGFR/Ras/PI3K/AKT axis → miR-9↓ → FOXP1↑	Glioblastoma	U87, U373 ΔEGFR cells	6
microRNA-34a	down	FOXP1 ↓	malignant transformation ↓	Myc↑ → miR-34a↓ → Foxp1↑	Gastric DLBCL	U2932	7
microRNA-376-a	down	FOXP2 ↓	cell proliferation↓ apoptosis↑	cyclin D2↓, cyclin A↓, Bax↑ and Bcl-2↓.	Lymphoma	JeKo-1	8
microRNA-139	down	FOXP2 ↓	proliferation↓migration↓	FOXP2 is a direct target of miR-139	Osteosarcoma	SAOS-2 MG63	9
microRNA-7 microRNA-155		induced by FOXP3	Transformation of the healthy breast epithelium to a cancerous phenotype↓	FOXP3 and FOXP3-regulated microRNAs → SATB1↓	Breast	BT549	12
microRNA-146		Induced by FOXP3	proliferation↓ apoptosis↑	FOXP3 → miR-146a/b↑→ NF-κB activation↓ by repressing Irak1 and Traf6	Breast	T47D, BT474, MDA-MB-468	10
microRNA-146		induced by FOXP3	apoptosis ↑ during tumor initiation tumor supression	FOXP3 → miR-146a/b↑→ NF-κB activation↓ by repressing Irak1 and Traf6	Prostate	PC3 DU145 LNCaP	11
microRNA-338-3p	down	FOXP4 ↓	proliferation↓ cell cycle arrest↑	miR-338-3p could directly target FOXP4	HCC	HepG2 Hep3B,QGY7703	13
microRNA-491-5p	down	FOXP4 ↓	proliferation↓ migration↓ invasion↓ apoptosis↑	FOXP4 is a target of miR-491-5p	Osteosarcoma	SAOS-2 MG63,U-2OS	14
microRNA-138	down	FOXP4↓	growth↓ invasion↓	miR-138 was the upstream regulator of FOXP4	NSCLC	SK-MES-1, A549, H460, SPC-A1	15.1/15.2
Oncogenic miRNAs							
microRNA-92a	up	FOXP1↓	cell proliferation↑cell cycle progression↑ tumor growth↑	FOXP1 was identified as a functional downstream target of miR-92a	OSCC	HSC3, OC3, SSC25 Tca-8113	16
microRNA-504	up (stage lll, lV)	FOXP1↓	invasion ↑ metastasis ↑	CTGF → miR-504↓ → FOXP1↑	OSCC	SAS	18
microRNA-19a	up	FOXP1↓	cell viability↑ colony formation↑ migration↑ invasion↑	miR-19a↑ → FOXP1, TP53INP1, TNFAIP3, and TUSC2↓	Lung	LK79	17
microRNA-196b	up	FOXP2 ↓	migration ↑ invasion ↑	miR 196b could directly bind to the 3'UTR of FOXP2mRNA	HCC	HCCLM3, Huh7 Hep3B, MHCC97H	19
microRNA-23a	up	FOXP2 ↓	proliferation↑ invasion↑	miR-23a directly targets FOXP2	PDAC	Aspc-1, Capan-2, Bxpc-3, Panc-1, MIA-Paca-2, SW1990	20
microRNA-190	up	FOXP2↓	invasion ↑ migration ↑proliferation↑	the direct target regulation of miR-190 to FOXP2	Gastric	GC tissue	21
microRNA-155	up	induced by FOXP3	Tumor initiaion	FOXP3 → BRCA1↓ → miR-155↑	Breast	Breast tissue	22
LNCRNAs							
MALAT1	up	FOXP1↑	proliferatin↑ cell grotwh ↑ apoptosis↓ G1/S phase ↓	MALAT1↑ → Foxp1↑ through sponging mir-509-5p	multiple myeloma	MM.1S, OPM-2, NCL-H929, U266, RPMI-8226	23
SNHG12	up	FOXP1↑	proliferation↑ apoptosis↓ cell growth↑ migration↑	SNHG12/miR-101-3p/FOXP1 axis	Glioma	U87 U251, A172, SHG44	24
UFC1	up	FOXP3↑	cell proliferation↑ migration↑ invasion↑ apoptosis↓	E2F1-linc-UFC1/miR-34a/FOXP3 axix	Cervical	Hela sila	25
7SL	up	inhibited by FOXP3	tumor growth	FOXP3 → 7SL↓ → P53↑ feedback loop	Breast	MCF-7 MCF10A	26
MFI2	up	FOXP4↑	proliferation↑ apoptosis↓ migration↑ invasion↑	correlation between MFI2 expression and FOXP4 expression	Osteosarcoma	MG63 SAOS-2	27
Circular RNAs							
Circ-SHKBP1	up	FOXP1↑ FOXP2↑	angiogenesis ↑	circ-SHKBP1 → miR-544a/miR-379 ↓ → FOXP1 FOXP2↑→ AGGF1↑→ PI3K/AKT ERK1/2 ↑	Malignant gliomas	GECs	28
CircRNAZNF609	up	FOXP4↑	proliferaion↑ invasion↑	CircRNAZNF609↑ → FOXP4↑ by sponging miR-138-5p	Renal carcinoma	A-498, ACHN	29
CircMYO9B	up	FOXP4↑	proliferaion↑ invasion↑ migration↑	CircMYO9B↑ → FOXP4↑ by sponging miR-4316	Breast	MCF-7 MDA-MB-231	30

**Fig. 4:**
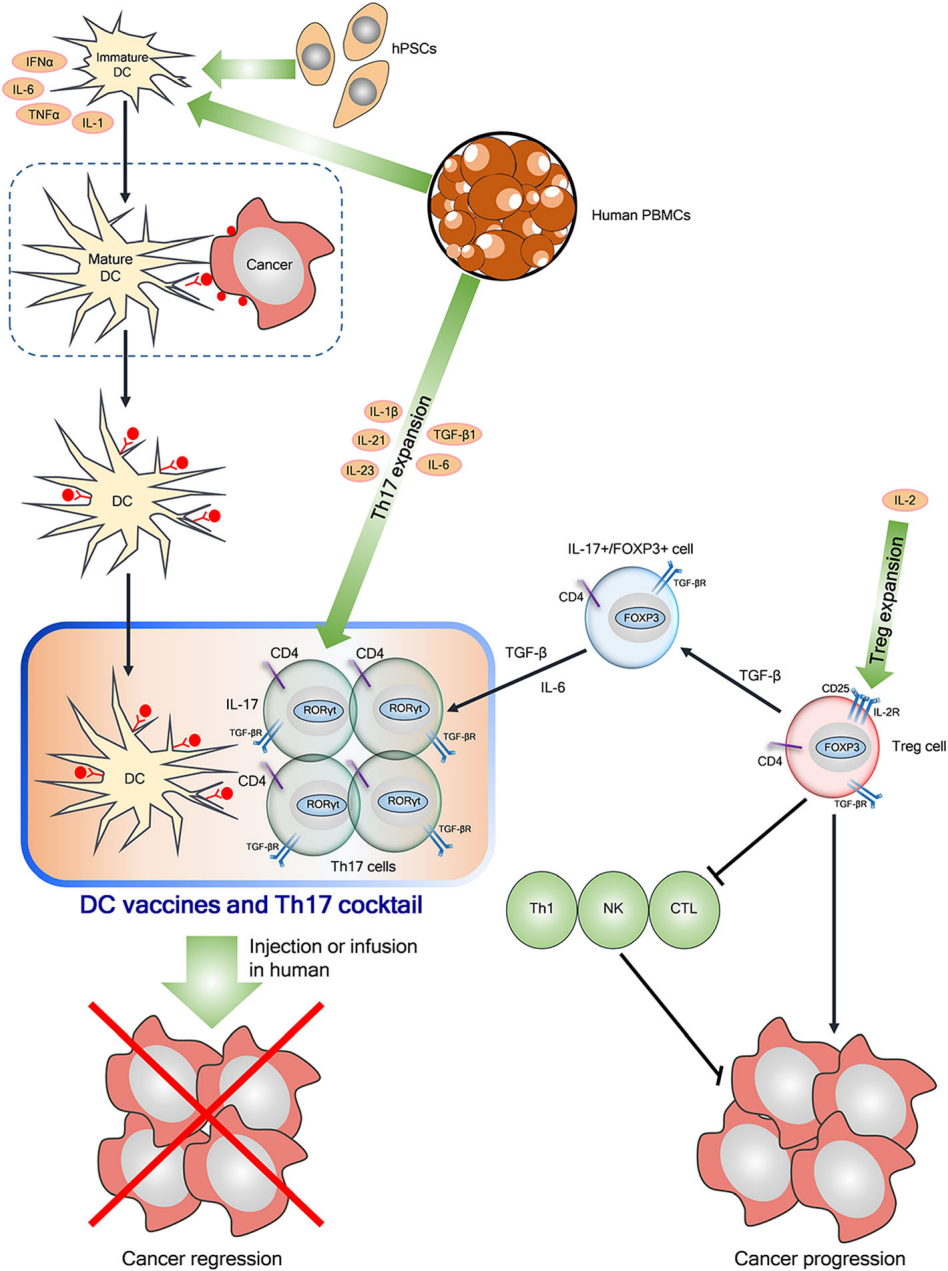
Cellular cancer immunotherapy by using Th17 cells and DC vaccine cocktail. Proinflammatory T helper 17 (Th17) cells, one of the CD4+ T cells, can produce IL-17 and protect cells against microbial infection, expressing RORγt (orphan nuclear receptor) [143], while excessive activation of Treg cells suppresses antipathogenic or anticancer immunity by inactivation of Th1, CTL and NK cells [220], leading to chronic infection and tumor progression [144]. Dendritic cells (DCs), the most efficient antigen-presenting cells (APCs) of the innate immune system, can be produced from peripheral blood mononuclear cells (PBMCs) or human pluripotent stem cells (hPSC) including embryonic stem cells and induced pluripotent stem cells [221]. Loading tumor specific antigens on immature DCs is the first step for DC vaccine production and DCs can be activated for maturation by defined cytokine formulation such as IL-1β^+^ IL-6^+^ PGE2^+^ TNF and TLR agonists (IL-2, IFNα/γ, GM-CSF, bacterial toxoids). Combination of TGFβ1 and IL-6 can be used for Th7 differentiation by reprogramming Treg cells into Th17 cells [146] and also a cocktail of TGFβ1, IL-6,IL-23, IL-1β and IL-21 is used for Th17 differentiation expansion from human PBMCs [218, 222]. Next generation cancer immunotherapy by a cocktail of DC vaccines and Th17 cells is suggested for cancer regression, which should be validated in vivo or clinically by intradermal injection or infusion after checking safety in the future

Additionally, FOXP3 is known a key transcription factor for the development and function of CD4^+^CD25^+^ regulatory T (Treg) cells. Recently the balance between FOXP3^+^ Treg cells and Th17 cells provides a new insight into a potent cellular cancer therapy, since FOXP3^+^Treg cells can be differentiated into antimicrobial Th17 cells or IL-17^+^FOXP3^+^ T cells to overcome the immunosuppressive function of Treg cells, leading to anti-tumor immunity in cancers. The efficacy of DC cancer immunotherapy has been limited due to immunosuppression by tumor-secreted TGF-β and Treg cells with tumor response rates rarely exceeding 15% in many clinical trials [213, 217], though many clinical trials were completed in melanoma (> 1000 patients), renal cell carcinoma (RCC; > 250 patients), glioblastoma (GBM; > 500 patients), prostate cancer (> 750 patients) [217]. Thus, Treg depletion and Th17 booster can be a potent strategy for DC cancer immunotherapy and mature DC vaccines were suggested as a next generation cancer immunotherapy with the standardization and quality of DC vaccines. Here we suggest the cocktail of specialized DC vaccines and Th17 cells by reprogramming Treg cells into Th17 cells [146] or ex vivo expansion of Th17 cells from human PBMCs [218] is suggested as a next generation cellular cancer immunotherapy, which should be further investigated in vivo and clinically (Fig. 4).

Overall, our review demonstrates that FOXP proteins are critically involved in cancer progression and immunology in concert with other molecules including noncoding RNAs and signaling pathways as potent biomarkers and targets for cancer diagnosis and treatment and also suggests another clinical trial for cellular cancer immunotherapy by DC vaccine and Th17 cells cocktail through Treg depletion, which should be also validated in vitro, in vivo and clinically in the future.

## Data Availability

Not applicable.

## Abbreviations

FHD: Forkhead DNA binding domain
FOXP: Forkhead box P
HEK293: Human embryonic kidney 293
IL-17: Interleukin 17
MALT: Mucosa-associated lymphoid tissue
MHC: Major Histocompatibility Complex
Myc: MYC proto-oncogene
NFAT1: Nuclear factor of activated T-cells 1
NF-κB: nuclear factor kappa-light-chain-enhancer of activated B cells
RUNX1: Runt-related transcription factor 1
STAT3: Signal transducer and activator of transcription 3
Th17: T helper 17
Treg: regulatory T
